# Cytoplasmic mRNA recapping has limited impact on proteome complexity

**DOI:** 10.1098/rsob.200313

**Published:** 2020-11-25

**Authors:** Bernice A. Agana, Vicki H. Wysocki, Daniel R. Schoenberg

**Affiliations:** 1 Center for RNA Biology, The Ohio State University, Columbus, OH 43210, USA; 2 Ohio State Biochemistry Program, The Ohio State University, Columbus, OH 43210, USA; 3 Department of Chemistry and Biochemistry, The Ohio State University, Columbus, OH 43210, USA; 4 Department of Biological Chemistry and Pharmacology, The Ohio State University, Columbus, OH 43210, USA

**Keywords:** RNA processing, proteomics, translation, protein expression, mRNA cap, cytoplasmic capping

## Abstract

The m7G cap marks the 5′ end of all eukaryotic mRNAs, but there are also capped ends that map downstream within spliced exons. A portion of the mRNA transcriptome undergoes a cyclical process of decapping and recapping, termed cap homeostasis, which impacts the translation and stability of these mRNAs. Blocking cytoplasmic capping results in the appearance of uncapped 5′ ends at native cap sites but also near downstream cap sites. If translation initiates at these sites the products would lack the expected N-terminal sequences, raising the possibility of a link between mRNA recapping and proteome complexity. We performed a shotgun proteomics analysis on cells carrying an inducible inhibitor of cytoplasmic capping. A total of 21 875 tryptic peptides corresponding to 3565 proteins were identified in induced and uninduced cells. Of these, only 29 proteins significantly increased, and 28 proteins significantly decreased, when cytoplasmic capping was inhibited, indicating mRNA recapping has little overall impact on protein expression. In addition, overall peptide coverage per protein did not change significantly when cytoplasmic capping was inhibited. Together with previous work, our findings indicate cap homeostasis functions primarily in gating mRNAs between translating and non-translating states, and not as a source of proteome complexity.

## 1. Introduction

The 5′ cap structure is a defining feature of all eukaryotic mRNAs and, for the vast majority of transcripts, its binding by eIF4E is the first committed step in translation initiation [1]. The cap is added co-transcriptionally [2,3] by the coordinated action of the bifunctional nuclear capping enzyme (RNGTT, termed CE), and the heterodimer of cap methyltransferase (RNMT) with its activating subunit RAM (or RAMAC) [4]. Capped analysis of gene expression (CAGE) is based on the 5′ cap as a functional marker for transcription start sites [5]; however, some of the earliest applications of CAGE identified capped ends downstream of transcription start sites and within spliced exons [6,7]. These findings coincided with the identification by our laboratory of a cytoplasmic population of CE that co-sedimented with a 5′-monophosphate kinase. The 5′-kinase converts RNA with a 5′ monophosphate end to one with a 5′ diphosphate, with subsequent GMP addition by CE resulting in a GpppX terminus [8] (reviewed in [9]). The 5′ kinase and CE assemble in the cytoplasmic capping complex by their respective binding to the second and third SH3 domains of adapter protein NCK1 [10], and the metabolon capable of converting 5′-monophosphate end to Cap 0 is completed by binding of the RNMT:RAM heterodimer to the N-terminus of cytoplasmic CE [11]. A recent proteomics analysis showed the cytoplasmic CE interactome is unexpectedly complex. Whereas nuclear CE interacts with four proteins [12] cytoplasmic CE interacts with 66 proteins, 52 of which are RNA-binding proteins [13]. This suggested target specificity is determined by the binding of one or more of these proteins, which in turn nucleate assembly of the cytoplasmic capping complex.

Functional analysis of cytoplasmic capping required the development of tools to selectively disrupt this process without affecting nuclear 5′ end processing. One approach targets cytoplasmic m7G cap methylation by overexpressing a truncated form of RNMT in the cytoplasm (ΔN-RNMT) with a mutation in the SAM binding site. ΔN-RNMT competes with cytoplasmic RNMT for binding to both CE and RAM, and its overexpression results in loss of mRNAs with improperly methylated caps [14]. Another approach targets GMP addition using an inducible form of CE with a mutation in the GMP binding site (K294). Like ΔN-RNMT this protein is restricted to the cytoplasm by loss of the nuclear localization domain and addition of the HIV Rev nuclear export signal. Both approaches provided support for recapping at native 5′ ends, and results from targeting cap methylation showed recapping can occur downstream within at least the 5′-untranslated region. This cytoplasmic cycling of caps off and back on (cap homeostasis) has been proposed as a way of gating mRNAs between translating and non-translating states [15,16]. While there is evidence for recapping downstream within the coding region [15,17,18], it remains to be determined if cytoplasmic mRNA recapping has a measurable impact on the proteome.

The current study addressed this using an isogenic system consisting of tetracycline-inducible U2OS cells stably transfected with a transgene expressing the K294A cytoplasmic guanylylation inhibitor [8,10,15,17,19]. The goal was to obtain a representative sampling that was of sufficient depth to determine if cytoplasmic capping impacts proteome complexity by looking for changes in protein and peptide representation after inhibiting this process. In addition, because tetracyclines have been reported to affect the metabolism and function of a number of human cell lines [20–22] we took advantage of having the parental tetracycline-inducible cell line to determine if treating cells with a tetracycline antibiotic has any impact on the proteome. Our findings show doxycycline treatment alone has minimal impact on the parental cell proteome. More importantly, inhibiting cytoplasmic capping had limited impact on the representative shotgun proteomics profile measured at 70–80% confluence, thus indicating this process is not a major contributor to proteome complexity.

## 2. Results and discussion

### 2.1. Doxycycline has minimal impact on the U2OS cell proteome

**Figure 1.**
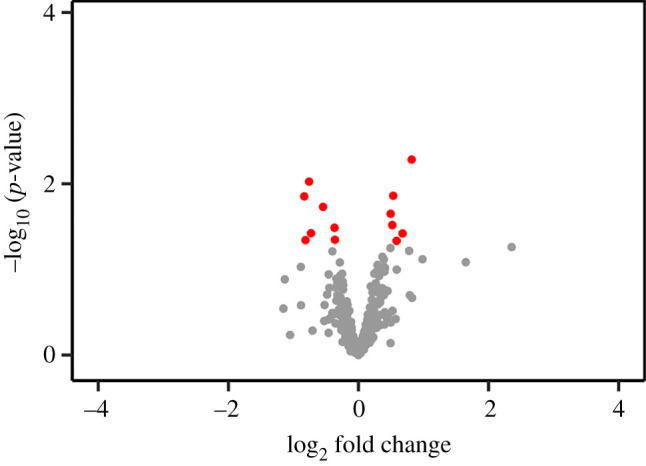
Doxycycline treatment has little impact on the proteome. Triplicate cultures of the parental U2OS-TR cells were treated for 24 h ± 1 µg ml^−1^ doxycycline. Protein lysate was reductively alkylated, trypsinized and analysed by LC-MS/MS. Differential peptide and protein expression was determined by comparing peptide spectral matches (PSMs) and filtering for false discovery rate (FDR) less than 0.01. The red dots indicate proteins that showed statistically significant changes.

The parental cell line for much of our work consists of U2OS osteosarcoma cells that stably express the tetracycline repressor protein (U2OS-TR cells [8]). That makes these cells a good model for testing whether the proteome is affected by treating cultured mammalian cells with tetracyclines. A proteome screen was performed on triplicate cultures of U2OS-TR cells that were treated for 24 h without and with this antibiotic (electronic supplementary material, table S1). A comparison of treated versus untreated cells showed doxycycline has minimal impact on the proteome of these cells (figure 1). There was a small increase in six proteins and similarly small decrease in seven proteins (electronic supplementary material, table S1), and these results are consistent with results from RNA-Seq of the same cells [14], which showed no impact of doxycycline on the transcriptome.

### 2.2. Cytoplasmic capping has limited overall impact on the proteome

As noted in the Introduction, one can inhibit cytoplasmic capping by interfering with GMP addition or with cap methylation. The current study employed the former approach, using a dominant-negative form of capping enzyme (termed K294A) to block the cytoplasmic guanylylation step. Twenty-four-hour induction of K294A causes prolonged inhibition of recapping that in turn results in the accumulation and/or loss of uncapped forms of cytoplasmic capping targets [15].

Total cellular protein was recovered from triplicate cultures of cells treated ± doxycycline, reductively alkylated, trypsinized and subjected to label-free shotgun proteomics as described in Material and methods. To increase the possibility of detecting quantitative changes in peptide representation, analysis was performed with a more sensitive mass spectrometer than that used to examine the effect of doxycycline on the parental cell line, coupled to improved front-end separation (UPLC and ion mobility). Differential peptide and protein expression were determined by comparing peptide spectral matches (PSMs) and filtering for false discovery rate (FDR) less than 0.01. This identified 21 875 peptides corresponding to 3565 proteins (electronic supplementary material, table S2); this represents about one-third of the total proteins that might be expressed by this cell line [23]. This number of proteins should be more than sufficient to draw conclusions on cap homeostasis. Note that we did not need or expect to measure the entire proteome of the cell line by the chosen shotgun proteomics approach. Moreover, since abundances vary over seven orders of magnitude total proteome measurements would require additional fractionation and analyses performed at multiple time points after doxycycline addition.

**Figure 2.**
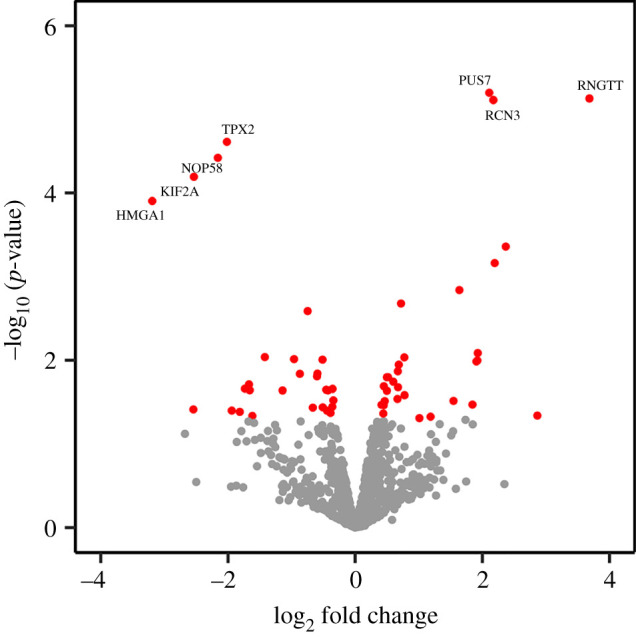
Inhibition of cytoplasmic capping has limited impact on the proteome. Triplicate cultures of tet-inducible U2OS cells carrying the dominant-negative K294A transgene (U2OS-K294A) were incubated for 24 h in medium ±1 µg ml^−1^ doxycycline. Cellular protein lysate was reductively alkylated, trypsinized and analysed as in figure 1 by LC-MS/MS but with a higher sensitivity instrument with improved front-end separation (UPLC coupled to ion mobility). The red dots indicate proteins that showed statistically significant changes. RNGTT corresponds to the induced K294A form of cytoplasmic capping enzyme.

Twenty-nine proteins (0.8% of 3565 proteins, plus the inhibitory form of RNGTT) showed a significant increase when cytoplasmic capping was inhibited (figure 2 and table 1). This is lower than the number of mRNAs that increased in [14], and may reflect both the shotgun proteomics approach, where the entire proteome is not captured, and more importantly, proteome buffering as described in [24]. The mRNAs that increased in [14] primarily encoded proteins involved in transcription and RNA processing, and we noted there, changes in these transcripts are likely a compensatory response to the decrease in a large number of other mRNAs as described in [25].

**Table 1.** Proteins that show significant change as a function of K294A inhibition of cytoplasmic capping. The proteins from figure 2 are listed in order of log_2_(fold change). RNGTT corresponds to the induced K294A cytoplasmic capping inhibitor.

upregulated proteins			downregulated proteins		
gene	Log_2_FC	*p*-value	gene	Log_2_FC	*p*-value
RNGTT	3.683235	7.42 × 10^−6^	HMGA1	−3.18987	0.000125
RPL36AL	2.863034	0.045837	MYH14	−2.54474	0.038705
PAPSS1	2.368596	0.000438	KIF2A	−2.53633	6.44 × 10^−5^
NCBP2-AS2	2.194125	0.00069	NOP58	−2.15911	3.81 × 10^−5^
RCN3	2.173334	7.75 × 10^−6^	TPX2	−2.01712	2.45 × 10^−5^
PUS7	2.108695	6.36 × 10^−6^	AGRN	−1.94046	0.03994
RNF114	1.927688	0.008204	DDX24	−1.81479	0.041356
LIN7C	1.921511	0.010086	ALDH1B1	−1.73369	0.021897
G45IP	1.906898	0.010342	NUP214	−1.66905	0.019458
AP2A1	1.84416	0.033842	PCBD1	−1.65788	0.022827
ARPC2	1.637564	0.001452	NPLOC4	−1.61658	0.046299
DCAF13	1.5456	0.030566	RBM42	−1.42023	0.009175
HIGD2A	1.184612	0.047272	ATAD2	−1.14108	0.022996
GPX1	1.008826	0.049148	CTNND1	−0.96172	0.0097
P4HA2	0.777128	0.026209	RBMX	−0.87146	0.014556
OXCT1	0.773697	0.009249	CDC5 L	−0.74805	0.00258
UBE2L3	0.721573	0.002094	LRRC47	−0.66754	0.036813
NUDC	0.6866	0.011318	DNAJA1	−0.60096	0.015585
DSG2	0.674596	0.021064	PRKDC	−0.5942	0.014467
QARS	0.671684	0.013567	RANBP2	−0.51412	0.00985
PLS3	0.665458	0.028991	LARP1	−0.51052	0.036478
TP53RK	0.599403	0.018085	SF3B2	−0.45578	0.022548
MVP	0.517951	0.015972	PPM1G	−0.44402	0.040009
PALLD	0.498868	0.015991	PTRF	−0.42853	0.0229
TRIP6	0.498671	0.02317	ETFA	−0.38873	0.042639
ACLY	0.465023	0.03075	PRDX2	−0.35913	0.03593
ACAT2	0.451171	0.020468	PPP5C	−0.35589	0.022009
OCIAD1	0.449599	0.03443	DPYSL3	−0.34331	0.030113
YWHAB	0.44258	0.04334			
CCDC50	0.415375	0.034067			

**Figure 3.**
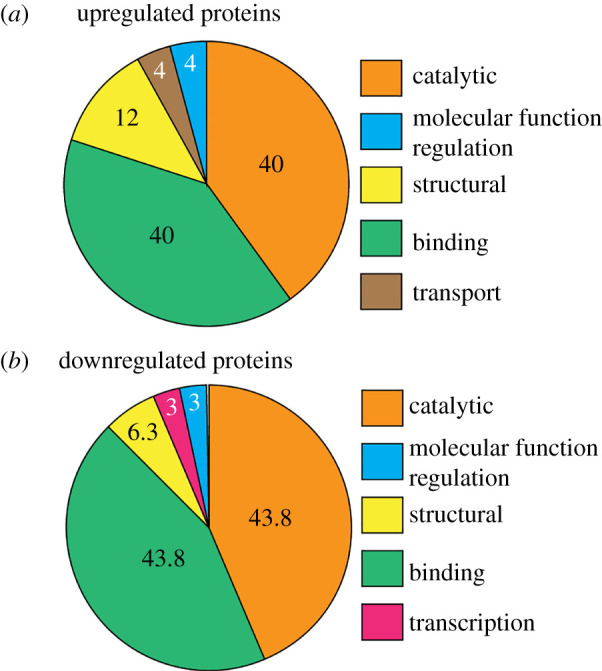
Gene ontology analysis of proteins that change following inhibition of cytoplasmic capping. (*a*) Gene ontology analysis of proteins that are upregulated in K294A-expressing cells compared to control. (*b*) Gene ontology analysis of proteins that are downregulated in K294A-expressing cells compared to control. The numbers in the pie charts indicate per cent representation of each set.

As one might expect from the experimental protocol, RNGTT (i.e. K294A) was the most highly induced protein. There was little evidence for functional relatedness between the proteins that increased in K294A-expressing cells, but it is not reasonable to draw conclusions on relatedness when only 0.8% of the proteins were increased. By Gene Ontology analysis, the increased proteins mostly fall under the broad categories of catalytic, binding, and structural proteins (figure 3*a*). A similarly small number of proteins (28, or 0.8%) were significantly decreased when cytoplasmic capping was blocked (figure 2 and table 1). Again, there was little evidence for functional relatedness between these. Gene Ontology analysis yielded groupings similar to those proteins that increased (figure 3*b*), but again one cannot draw conclusions based on such a small number of differentially expressed proteins. None of these proteins correspond to transcripts in [15] whose cap status changed following K294A induction. We suspect this is due to differences in the relative amount of uncapped RNA for any given transcript.

### 2.3. Peptide representation is unchanged by inhibition of cytoplasmic capping

**Figure 4.**
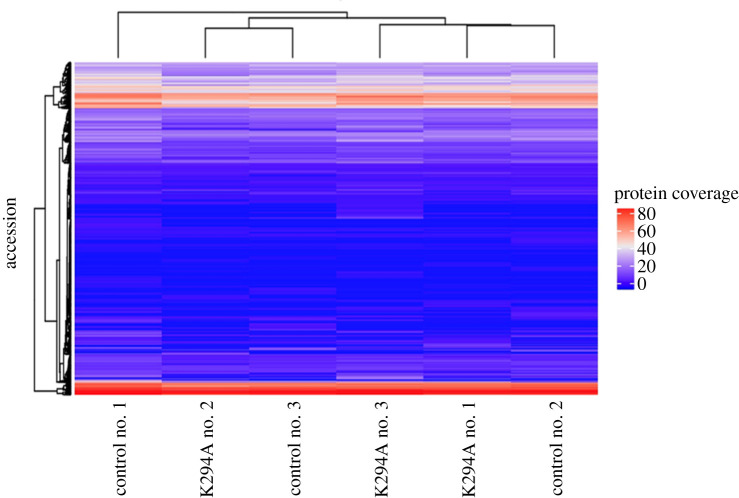
Peptide coverage is unchanged by cytoplasmic capping inhibition. Peptide coverage for each of the proteins in electronic supplementary material, table S2 was compared between protein samples for each of the control cell populations and cells treated with doxycycline to induce the K294A cytoplasmic capping inhibitor. Heatmap was generated using the R package ComplexHeatmap to reveal potential patterns in protein sequence coverage at the peptide level.

In [26] and [9], we put forward the idea that one function for cytoplasmic capping might be to expand the proteome. This was based on several findings. Approximately 25% of capped ends are located downstream of transcription start sites [6,7], and many of these lie upstream of potential start codons. There is also evidence from positional proteomics for N-termini that map downstream of canonical initiation sites [27–29]. While some of these correspond to known alternative initiation sites, propeptides and signal peptide cleavage sites, for many the origin remains unknown. Lastly, a number of mRNA targets acquire uncapped 5′ ends in the vicinity of known downstream cap sites when cytoplasmic capping is blocked [17,18]. Because there was good peptide coverage across most of the identified proteins, we reasoned that downstream initiation events would be evident by changes in peptide representation in control versus K294A expressing cells, with the most notable change being an increase in peptides nearest the native N-terminus. That turned out not to be the case. A comparison of peptide coverage between individual replicates in figure 4 showed no evidence for significant changes as a function of K294A expression. Thus, our results indicate that cytoplasmic capping has little overall impact on the proteome.

In summary, the accumulated data do not support a role for cytoplasmic capping in proteome complexity, but instead support the model of cap homeostasis put forward in [15], where decapping and recapping serves as a gating mechanism controlling the translation of a portion of the transcriptome. This is consistent with the observed recapping of RPS3, RPS4X and RPL8 mRNAs on their native 5′ ends [14], and with results of *in vivo* single-molecule translational dynamics that showed cycling of mRNAs between translating and non-translating states [16,30]. The only other known function of mRNA recapping also involves translation, as recapping within the 5′-UTR (e.g. EIF3D, EIF3 K) can change secondary structures and binding sites for regulatory proteins.

## 3. Methods

### 3.1. Cell culture and protein extraction

Tetracycline-inducible U2OS (U2OS-TR) cells and tetracycline-inducible U2OS cells stably transfected with pcDNA4/TO/myc-K294DNLS + NES-Flag (U2OS-K294A) were described previously [8]. Cells were maintained in a humidified incubator at 37°C under 5% CO_2_ and were discarded after no more than 10 passages. Cells were grown in McCoy's 5A medium (Thermo Fisher 116600) supplemented with 10% tetracycline-free fetal bovine serum (FBS, Atlanta Biologicals S10350). Triplicate cultures of parental U2OS-TR or K294A-expressing cells at 70–80% confluence were switched to medium without or with 1 µg ml^−1^ of doxycycline for 24 h. Prior to harvest cultures were washed three times with phosphate-buffered saline (PBS) and lysed using ice-cold lysis buffer (0.1 M HEPES, pH 8.5, 6M guanidine hydrochloride supplemented with one tablet of protease inhibitor (cOmplete Mini EDTA-free cocktail, Roche Life Science) and one tablet of phosphatase inhibitor (PhosSTOP, Roche Life Science). The cell lysates were sonicated using Sonic Dismembrator Model 100 (Fisher Scientific) for three cycles of alternating 30 s bursts followed by 30 s rest followed by centrifugation at 16 000× g for 15 min at 4°C. The protein concentration of the collected supernatant was determined by a bicinchoninic acid (BCA) protein assay kit (ThermoFisher Scientific).

### 3.2. Sample preparation for shotgun proteomics analysis

Four hundred milligrams of lysate was reductively alkylated by first incubating for 1 h at 37°C with 10 mM dithiothreitol, followed by 30 min alkylation (in the dark) at 25°C with 55 mM iodoacetamide (Sigma Aldrich). Samples were diluted sixfold with 50 mM ammonium bicarbonate to reduce the concentration of guanidine hydrochloride to less than 1 M. Tryptic digestion was performed by adding 2 µl of 1 µg µl^−1^ trypsin (1 : 200 w/w) supplemented with 1 µl of 1% ProteaseMAX surfactant (Promega) and incubating at 37°C for 3 h. Trypsin was inactivated by addition of trifluoroacetic acid (TFA) to a final concentration of 0.5%. The digestion products were centrifuged 16 000*g* for 10 min, and the supernatants were collected and evaporated to dryness.

### 3.3. Protein analysis by shotgun proteomics

#### 3.3.1. Impact of doxycycline: Orbitrap Elite

Analysis of the impact of doxycycline on the U2OS cell proteome (U2OS-TR, figure 1*b*) was performed by LC-MS/MS on a Waters nanoACQUITY UHPLC system (Waters Corporation, Milford, MA) coupled to a Thermo LTQ-Orbitrap Elite hybrid mass spectrometer via an Easy Spray ion source (Thermo Fisher Scientific, Bremen, Germany). Peptides (0.5 µg) reconstituted in 0.1% formic acid was injected and loaded onto a Waters Symmetry C18 trap column (100 Å, 5 µm particle diameter, 180 µm × 20 mm) for desalting at a flow rate of 20 µl min^−1^. The analytical separation was achieved on a C18 reversed-phase column (75 µm × 15 cm, PepMap C18, 3 µm, 100 Å) at pH 2.4 which was equilibrated to initial conditions of 98% (v/v) A1 and 2% (v/v) B1. The subsequent separation was achieved at 35°C where the % B1 was maintained at 2% for 5 min; 2–35% over 75 min; 35–45% over 10 min and 45–85% over 10 min at a flow rate of 0.3 µl min^−1^. The column was held at 85% (v/v) B1 for 5 min before reaching initial conditions after 10 min. The heated capillary temperature and electrospray voltage on the Orbitrap Elite were 200°C and 1.5 kV, respectively, using top 15 data-dependent acquisition in positive ion mode. The MS scans were acquired at a resolution of 120 000 with a automatic gain control (AGC) target value of 1 × 10^6^ for a scan range of 400–1600 *m/z*. Collision-induced dissociation (CID) spectra were obtained in the ion trap with AGC target of 1 × 10^4^, maximum ion injection time (IT) of 50 ms, 1 *m/z* isolation width, normalized collisional energy (NCE) of 35 and ion activation time of 10 ms. The transfer tube S-lens RF was 49% and dynamic exclusion was set at 15 s with a repeat count of 1 for an exclusion list size of 500.

#### 3.3.2. Impact of cytoplasmic capping: timsTOF Pro

Samples from U2OS-K294A cells were analysed using a nanoElute coupled to a timsTOF Pro equipped with a CaptiveSpray source (Bruker, Germany). Peptides (0.2 µg) was separated on a 25 cm × 75 µm analytical column, packed with 1.6 µm C18 beads (IonOpticks, Australia). The column temperature was maintained at 50°C using an integrated column oven (Sonation GmbH, Germany). Separation was achieved using 0.1% formic acid (A1) and acetonitrile with 0.1% formic acid (B1) as mobile phases. The column was equilibrated with four column volumes of 100% solvent A1 before loading sample at a maintained pressure of 800 bar. Peptide separation was achieved at 0.4 ml min^−1^ using a linear gradient from 2% to 25% solvent B1 over 90 min, 25% to 37% over 10 min, 37% to 80% over 10 min and maintained for 10 min for total separation method time 120 min. Data acquisition on the timsTOF Pro used the parallel accumulation serial fragmentation (PASEF) acquisition mode. Instrument settings included default imeX mode, mass range 100 to 1700 *m/z*, capillary voltage of 1.6 kV, dry gas 3 l min^−1^ and dry temp of 180°C. PASEF settings included 10 ms ms^−1^ scans at 1.18 s total cycle time, scheduling target intensity of 20 000, charge range 0–5, active exclusion release after 0.4 min, and CID collision energy 42 eV.

## 4. Data processing and analysis

### 4.1. Database search for shotgun proteomics

The data files from the timsTOF Pro were converted into mgf-files using MSConverGUI (ProteoWizard). Protein identifications for the Orbitrap Elite and timsTOF Pro data were obtained via the Thermo Proteome Discoverer software (v. 1.4.1.14) using the Sequest search algorithm and the Uniprot Swissprot canonical *H. sapiens* proteome (as of 15 June 2018 with 20 292 entries). Search parameters included precursor mass tolerance of 10 ppm and 50 ppm for the Orbitrap Elite and timsTOF Pro, respectively, and fragment mass tolerance of 0.6 Da and 0.05 Da, respectively. Database searches used trypsin (full) cleavage with a maximum of 2 missed cleavages. Cysteine carbamidomethylation was set as a fixed modification while oxidation of methionines and protein N-terminal acetylation were set as variable modifications. Percolator was used for estimation of the PSM (peptide spectrum match) level FDR of peptide identification. Proteins were identified at a 0.01 FDR for PSM and 0.01 protein FDR. Protein groups were filtered only to include peptides with 99% confidence.

### 4.2. Statistical analysis

Label-free relative quantification was carried out using the Limma package in R [31] using peptide spectral matches (PSMs) or spectral counts. Differential expression of individual proteins was determined using proteins with at least two PSMs and two unique peptides. Data were filtered for PSMs observed in all replicates of at least one condition and normalized using quantile normalization. PSMs were log_2_ transformed and differential enrichment analysis was carried out using linear models combined with empirical Bayes statistics function in Limma. False discovery correction was applied using the Benjamani-Hochberg method and data were visualized using volcano plots with significant *p*-values ≤0.05 and heatmaps.

## 5. Gene ontology analysis

Gene ontology analysis was performed using PANTHER (Protein ANalysis THrough Evolutionary Relationships) Classification System [32,33], v. 15.0 released 2020-02-14. Functional classification of significant proteins was searched against the PANTHER Classification System using their corresponding gene names with *Homo sapiens* as the select organism. Pie chart illustrations of gene ontology annotations for molecular function of proteins with significantly different expression are presented in figure 3.

## Data accessibility

The complete mass spectrometry dataset is available from the ProteomeXchange Consortium via the jPOST partner repository under the dataset identifier PXD016907.
